# Hospital Cluster of HBV Infection: Molecular Evidence of Patient-to-Patient Transmission through Lancing Device

**DOI:** 10.1371/journal.pone.0033122

**Published:** 2012-03-06

**Authors:** Simone Lanini, Anna Rosa Garbuglia, Vincenzo Puro, Mariacarmela Solmone, Lorena Martini, William Arcese, Alessandro Nanni Costa, Piero Borgia, Pierluca Piselli, Maria Rosaria Capobionchi, Giuseppe Ippolito

**Affiliations:** 1 National Institute for Infectious Diseases “Lazzaro Spallanzani”, Rome, Italy; 2 Fondazione Policlinico “Tor Vergata”, Rome, Italy; 3 CNT ISS-Centro Nazionali Trapianti, Rome, Italy; 4 Agenzia di Sanità Pubblica del Lazio, Rome, Italy; Instituto de Higiene e Medicina Tropical, Portugal

## Abstract

**Introduction:**

In western countries the transmission of hepatitis B virus (HBV) transmission through multi-patients lancing devices has been inferred since early ‘90s, however no study has ever provided biological evidence which directly link these device with HBV cross-infection. Here we present results of an outbreak investigation which could associate, by molecular techniques, the use of lancing device on multiple patients with HBV transmission in an Italian oncohematology unit.

**Methods:**

The outbreak investigation was designed as a retrospective cohort study to identify all potential cases. All cases identified were eventually confirmed through molecular epidemiology techniques. Audit of personnel including extensive review of infection control measures and reviewing personnel's tests for HBV was done identify transmission route.

**Results:**

Between 4 May 2006 and 21 February 2007, six incident cases of HBV infection were reported among 162 patients admitted in the oncohematology. The subsequent molecular instigation proved that 3 out 6 incident cases and one prevalent cases (already infected with HBV at the admission) represented a monophyletic cluster of infection. The eventual environmental investigation found that an identical HBV viral strain was present on a multi-patients lancing device in use in the unit and the inferential analysis showed a statistically significant association between undergoing lancing procedures and the infection.

**Discussion:**

This investigation provide molecular evidence to link a HBV infection cluster to multi-patients lancing device and highlights that patients undergoing capillary blood sampling by non-disposable lancing device may face an unacceptable increased risk of HBV infection. Therefore we believe that multi-patients lancing devices should be banned from healthcare settings and replace with disposable safety lancets that permanently retract to prevent the use of the same device on multiple patients. The use of non-disposable lancing devices should be restricted to individual use at patients' home.

## Introduction

In western countries cross infections of hepatitis B virus (HBV) have been mainly reported as a consequence of failure to apply standard measures for infection control [1]. In 2009 we published a systematic review including 33 HBV outbreaks which occurred in EU and USA healthcare settings (HCS) [2] and we found that the use of multi-patient lancing devices for capillary blood sampling was the second most frequent route of HBV transmission after multi-use drug vials; similar findings were also reported by a similar review published in the same year [3]. In fact, the role of multi-patient lancing devices in HBV transmission has been inferred since early ‘90s [2], [4]–[6], however no study has ever provided biological evidence to link multi-patient lancing devices with HBV cross infection.

During February 2007 3 cases of acute HBV infection occurred in a single Italian oncohematology unit over less than 2 months. On March 2007, the INMI-Lazzaro Spallanzani (INMI) epidemiology team was asked to investigate the event and, if epidemic cluster(s) were confirmed, to identify and remove potential causes. This report presents the results of the outbreak investigation, which provide molecular evidence to link the use of a single multi-patient lancing device to the transmission of HBV within the oncohematology unit. The report has been written according to the ORION statement [7].

## Materials and Methods

### Ethical statement

All data contained in the manuscript are obtained during the epidemiological investigation performed in order to identify/contain an ongoing epidemic cluster among frail subjects, to provide recommendations, to prevent new outbreaks and to avert complications in infected subjects. For the purpose of the current publication there was no information that could identify the patient personally.

The approval of INMI Spallanzani's IRB was not required since we operated under emergency circumstance (i.e.: potential risk of death of already infected subjects due to complications and the risk of further spreading of the infection) and patients never underwent individual intervention for the purposes of this study but only according to their needs and clinical judgment. Individual written consents was obtained for all subjects who were included in the case-control and/or provided biological specimen(s) provided that they were still alive when specimen(s) were obtained.

### Study Design

Based on HBV's incubation period and the time of infection onset, we define a historical cohort to include all patients who had been admitted to the oncohematology unit between 4 May 2006 and 21 February 2007. In addition, a prospective surveillance period was performed between march 2007 and march 2008 (see table 1) to confirm the end of the transmission.

**Table 1:** Shows all the interventions performed to contain the spreading of the infection either by hospital authority before the INMI's involvement or undertaken by INMI epi-team its-self.

Institution responsible for the activity	Type of Activity	Date
Local health authority	Deferral of HSC autografts	1 March to 1 April 2007
	Map of blood donor	10 March 2007
INMI's Epi-team	Acquisition of data about all interventions already undertaken	10 March 2007
	Timing meeting between hospital authority and epi-team (5 meeting held)	10 March to 22 June 2007
	Test of all HSC unit to restart autografts	15–20 March 2007
	Auditing: Interview of medical heads of OHU, TMU and IRU	10–15 March 2007
	Auditing: Interview of OHU's nurse and nurse coordinator	19–23 March 2007
	Auditing: Acquisition of internal protocol in use in OHU, TMU and IRU	15 march 2007
	Auditing: Review of HCW's HBV testing	April 2007
	Environmental inspection in OHU	2 April 2007
	Environmental inspection in TMU	15 March 2007
	Implementation of a enhanced survey to detect new cases of HBV infection	March 2007–March 2008
	Educational event on blood-borne infection (2 days course for OHU's HCW)	14–15 June 2007

**INMI** = National Institute for Infectious diseases; **HSC** = hematopoietic stem cells; **HBV** = hepatitis B virus; **HCW** = healthcare workers; **OHU** = onco-hematology unit; **TMU** = transfusion medicine unit; **IRU** = interventional radiology unit.

To assessed the statistical association between potential risk factors and HBV infection we carried out a case control study nested into the historical cohort (i.e. nested case control study). Case finding was performed by reviewing clinical charts, testing patients' biological specimens preserved in the hospital and testing living patients at least 6 months after their last admission to oncohematology unit.

Molecular epidemiology techniques were used to confirm cases.

### Definitions

#### Pre-admission HBV-status

Susceptible: a patient testing negative for anti-HBcAg and/or anti-HBsAg markers at any time after the first admission to oncohematology unit.

Non-susceptible: a patient who, before first admission or within 14 days after first admission to oncohematology unit, tested either:

- Previously infected: anti-HBcAg and/or HBVDNA and/or HBsAg positive;
- Vaccinated: anti-HBsAg positive and anti-HBcAg negative.

Undefined: a patient who was never tested.

#### Case definition

Prevalent case: a case who was already admitted as previously infected.

Incident case: a case who was susceptible at pre-admission and eventually became previously infected.

Index case: a prevalent case infected with a HBV molecular variant identical to confirmed case(s).

Confirmed case: an incident case infected with a HBV molecular variant identical to the index case.

Suspect case: an incident case for whom a HBV molecular variant was not defined.

Excluded cases:

- all patients “non-susceptible” at pre-admission apart form index case(s);
- all patients who were susceptible/vaccinated at least 6 months after the last admission to oncohematology unit;
- all patients who were infected with a HBV molecular variant different from the variant(s) infecting confirmed/index case(s).

Not assessable: all patients who did not meet any of the above definitions.

### Setting

The outbreak occurred in a medium size public general hospital (about 750 beds). The investigation involved 3 hospital units including.

1. The oncohematology unit used to care for patients with and without cancer, was capable of autologous hematopoietic stem cell transplant and could accommodate a maximum of 18 patients in 7 rooms (3 of which were single-bedded).
2. The transfusion medicine unit consisted in a three-room ward and provided the oncohematology unit with transfusion and hematopoietic stem cell apheresis/transplant service.
3. The interventional radiology unit consisted in an operating theatre with dedicated personnel within the radio-diagnostic department. Patients admitted to oncohematology unit used to be sent to the interventional radiology whenever they needed central venous catheter insertion.

### Interventions

When the epidemiology team was formally involved in the investigation several interventions had already been undertaken by local authority. Table 1 reports the time-table of interventions undertaken before and after the formal initiation of the investigation.

By mid March 2007, one-year enhanced surveillance for viral hepatitis for one year was implemented. This consisted in testing all patients admitted to oncohematology unit for anti-HBsAg, anti-HBcAg and HBsAg at admission and whenever they showed signs of acute hepatitis (i.e. ALT>80 UI). A serum sample was sent to INMI for all patients positive for HBsAg. Audit was carried out to identify and remove potential gaps in infection control measures. All internal protocols were assessed and oncohematology unit's nurses were interviewed using a predefined form, while the medical heads of all the three units were informally asked about the general procedures of their own unit. The potential role of HCW-to-patient transmission was evaluated by reviewing HCW's tests for HBV, as preformed annually. An environmental investigation was conducted to evaluate the role of the environment in the spreading of HBV. The Oncohematology unit and the transfusion medicine unit were inspected and environmental samples were obtained. In addition we assessed (by direct observation) the actual implementation of infection control measures as reported in the internal protocols.

### Virology

#### HBV serology and HBV-DNA testing

Standard serum samples: HBsAg, anti-HBsAg, and total anti-HBcAg were evaluated using quantitative enzyme immunoassay (Axsym, Abbott Diagnostics, Wiesbaden, Germany). HBV DNA was measured using Cobas Ampliprep/Cobas TaqMan HBV assay (Roche Diagnostics, Basel, Switzerland).

Lancing Device: the internal and external surfaces of the multi-patient lancing device was washed with heat inactivated fetal calf serum and the elute was eventually tested for HBV DNA by Cobas Ampliprep/Cobas TaqMan.

Liver biopsies: paraffin was removed using a progression of xylene and ethanol washes [8] and homogenized tissue was processed by QIAamp DNA Minikit (QIAGEN, Hilden, Germany). The extracted DNA was tested for HBV-DNA presence.

Bone marrow: HBV-DNA from bone marrow was extracted using QIAamp minikit (QIAGEN, Hilden, Germany) and employed for HBV DNA detection.

Blood samples derived from apheresis: plasma was separated from PMBCs by centrifugation, screened for HBsAg, total anti-HBcAg and, if positive, HBV-DNA molecular testing was performed.

Cryopreservation tank: detritus was collected and allowed to thaw. The extracts were tested for HBV DNA detection.

#### Molecular analysis of HBV DNA

Amplification of two region of HBV viral genome were performed as previously described [9]–[10]; this is the polymerases and the core/precore regions. In particular, 558 nucleotides (nucleotides 345–902) and 562 nucleotides (nucleotides 1794–2355) were sequenced for polymerase and core promoter/precore gene respectively. The nucleotide numbers are in accordance with a genotype D HBV isolate of 3182 nucleotides [AB205127]. Surface region of HBV extracted from the multi-patients device was cloned into the donor vector of the Gateway cloning system (Life Technologies) according to manufacturer's instruction. PCR-amplified HBV-DNA and seven randomly selected clones were sequenced directly on the automated ABI Prism 3100 sequencer, using the BigDye Terminator cycle sequencing kit (Applied Biosystems, Warrington, UK). Phylogenetic trees were constructed using the neighbor-joining method, including HBV reference sequences from GenBank, as well as sequences of genotype D obtained from routine Laboratory samples. All the algorithms used are included in the Mega package (version 2.1). The results of this analysis were used to confirm a monophyletic cluster of infection. Bootstrap analysis with 1,000 replications was performed to assess the significance of the nodes; values >85% were considered to be significant. The sequence data from the current report have been submitted GenBank with the accession numbers–JQ403585–JQ403598 (polymerase region sequence) andJQ403599–JQ403606(core/precore sequence).

### Statistical methods

Statistical association was studied by a nested case control study. Cases were all subjects who met the definition of “incident case” and controls were all subjects who tested negative for anti-HBcAg 6 months after the end of the last admission. The list of potential risk factors was defined according to published data [2] and the results of the audit. This is: gender (binary variable); age (in years, continuous variable); being admitted for cancer (binary variable); having underwent hematopoietic stem cell apheresis and/or transplant (binary variable), having underwent any kind of surgery since the first admission (binary variable); having received blood transfusion while admitted (binary variable); having had at least one day of central venous catheterization while admitted (binary variable); median exposure to multi-patient lancing device while admitted with the index case and either until the onset of acute hepatitis or the first positive anti-HBcAg test (in days, continuous variable). Diagnosis of acute hepatitis was according to clinical records (i.e. clinical diagnosis of acute hepatitis eventually confirmed by laboratory test, as reported in patients' charts).

Association of the outcome to binary variables was assessed with Fisher's test; relative odds ratio (OR), 95% confidence intervals (95%-CI) and p-values were provided. Association of the outcome to continuous variables was assessed with the Mann-Whitney U test and relative medians values, inter-quartiles ranges (IQR) and p-values were provided.

Analysis was performed using Stata Statistical software, version 11.2 (StataCorp, Texas, USA).

## Results

### Cohort study and nested case control

Between 4 May 2006 and 21 February 2007 the oncohematology unit performed a total of 272 admissions on 162 individual patients. We could define the HBV pre-admission status for 83 out of 162 patients; of these 33 were non-susceptible (including 3 HBsAg positive prevalent cases) and 50 were susceptible. No information about HBV were available for the other 79 patients (58 died before start of investigation, 21 were lost, 6 refused to provide serum samples). Among the 50 patients susceptible at admission, 6 incident cases were identified (3 HBsAg positive, 2 anti-HBsAg/anti-HBcAg positive, and 1 isolate anti-HBcAg positive).

**Figure 1:**
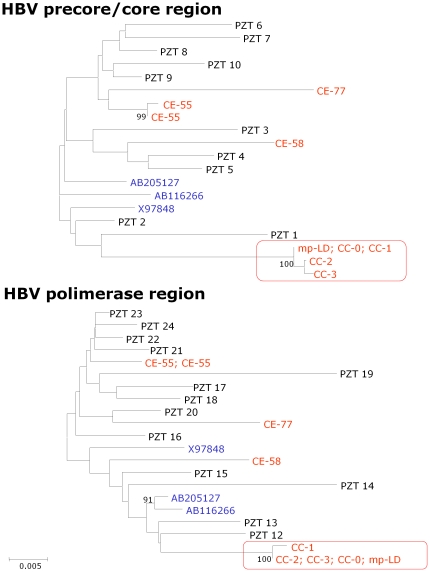
Phylogenetic tree of HBV-precore/core and HBV-polymerase region. The analysis was performed on 3 out 6 incident cases, 2 out 3 prevalent cases, 2 additional cases identified during the enhanced surveillance period and on elute from the mp-LD (red codes). The phylogenetic trees also include 10 (precore/core) and 13 (polymerase) sequences of the genotype D HBV from patients not related to the outbreak who were referred to the laboratory for diagnostic purpose (black codes) and 3 genotype D sequences from GenBank (blue codes). The analysis shows that all patients within the study were infected with a genotype D HBV. In addition one prevalent cases (index case; CC-0), 3 incident cases (confirmed cases; CC-1, CC-2 and CC-3) and the elute from multi-patients lancing device (mp-LD) where infected with a highly related HBV molecular variant. In fact, these molecular variants form a monophyletic cluster distinct from the other sequences by very high bootstrap value (red box). In contrast one prevalent case (excluded case CE-58) and the 2 cases (excluded cases; CE-55 and CE-77) detected during the enhanced surveillance were infected with unrelated HBV molecular variants. The epidemiologically unrelated cases, both form our laboratory archive and from GenBanK, were infected with genetically distant variants as expected. Boxes indicate the epidemic cluster; the bars indicate the genetic distance.

**Figure 2:**
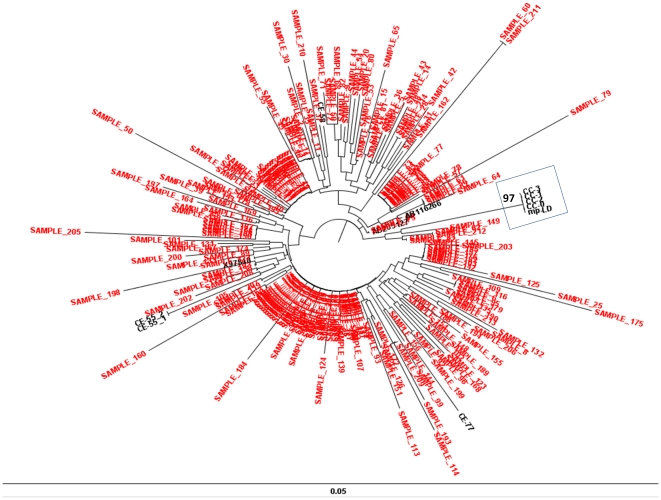
Extended phylogenetic analysis for HBV polymerase region. Extended phylogenetic tree including 214 genomic sequence of HBV-polymerase region. In red unrelated HBV molecular variant which were obtained from patients referred to our laboratory in about 7 years. In black: 3 genotype D sequences from GenBank (AB205127; AB116266; X97848); 3 excluded case form our investigation (CE-55_1/2; CE-58; CE-77); The index case (CC-0); 3 confirmed cases (CC-1; CC-2; CC-3); the elute form multi-patients lancing device (mp-LD). Even after this new analysis CC-0, CC-1, CC-2, CC-3 and the elute from mp-LD formed a monophyletic cluster distinct from the other sequences by very high bootstrap value. The box indicates the epidemic cluster; the bars indicate the genetic distance.

Two out 3 prevalent cases and 3 out 6 incident cases underwent molecular investigation to type HBV variant. The characterization of the HBV molecular variants (figure 1) showed that one prevalent cases (index case; code CC-0 in figure 1 and table 2) and 3 incident cases (confirmed cases; code CC-1, CC-2, CC-3 in figure 1 and table 2) were infected with an identical HBV variant. The other prevalent case was infected with an unrelated HBV variant (excluded case; code CE-58 in figure 1). As the genotype D is the most prevalent among HBsAg positive subjects in Italy, to increase discriminatory power of the molecular investigation, we produced a subsequent phylogenetic tree using 214 HBV-polymerases genomic sequence from unrelated subjects obtained during routine clinical practice. Even after this extended analysis CC-0, CC-1, CC-2, CC-3 and the elute from mp-LD formed a monophyletic cluster distinct from the other sequences by very high bootstrap value (figure 2).

**Table 2:** Shows main clinical feature of incident case (CCs codes are for confirmed and case CDs codes are for suspected cases).

Case's features	CC-0	CC-1	CC-2	CC-3	CD-4	CD-5	CD-6
Case definition	index	confirmed	confirmed	confirmed	potential	potential	potential
HBV molecular variant					NT	NT	NT
Sex	M	F	M	F	F	M	M
Age in years	66	58	71	40	80	52	56
Diagnosis	MM	MM	NHL	NHL	AML	MM	NHL
First admission	11/05/06	08/07/06	22/05/06	09/05/06	21/10/06	18/09/06	08/11/06
Last negative anti-HBcAg	none **[** a **]**	10/10/06	14/07/06	01/01/07	23/10/06	01/04/07	08/11/06
First positive anti-HBcAg	none **[** a **]**	15/01/07	12/02/07	01/03/07	15/01/08 **[** b **]**	12/05/08 **[** b **]**	09/10/07 **[** b **]**
Days of Exposure to mp-LD	159	28	31	35	15	3	31
Transfusion	No	No	Yes **[** c **]**	No	Yes **[** c **]**	No	No
Surgery	Yes	Yes	Yes	Yes	Yes	Yes	Yes
HSC autograft	Yes	No	Yes	Yes	No	Yes	Yes
CVC	Yes	No	Yes	Yes	No	Yes	Yes

**M** = male; **F** = female; **MM** = multiple myeloma; **NHL** = non-Hodgkin lymphoma; **AML** = acute myeloid leukemia; **mp-LD** = multi-patients lancing device, **HSC** = hematopoietic stem cells; **CVC** = central venous catheter.

a ) Positive before the first admission.

b ) Patient never reported symptoms of acute hepatitis and were found to be positive during test performed 6 months after the last admission to oncohematology unit.

c ) No common donor between patients was found.

One prevalent case refused to provide serum sample and was no further investigated. Three incident cases did not underwent molecular investigation either because are already anti-HBsAg positive or had undetectable HBV DNA (suspect cases CD-4, CD-5, CD-6 in table 2).

The review of admission records confirm that the all 6 incident cases met and underwent capillary blood sampling along with the index case. In addition the results of the nested case control study including the 50 susceptible patients (6 cases and 44 controls) provided good evidence of association between the duration of exposure to multi-patient lancing device while admitted with the index case and being an incident case (table 3).

**Table 3:** Nested case control study for analysis of association between being incident case and potential risk-factor.

Risk factors		Case (n = 6)	Control (n = 44)	OR (95%CI)	P-value
Gender (%) **[** a **]**	*male*	3	20	1	
	*female*	3	24	1.20 (0.14–9.95)	1.0000
Cancer (%) **[** a **]**	*yes*	6	37	1	
	*no*	0	7	na	0.5760
HSCT (%) **[** a **]**	*yes*	4	14	1	
	*no*	2	30	4.29 (0.52–51.04)	0.1710
Surgery (%) **[** a **]**	*yes*	6	34	1	
	*no*	0	10	na	0.3271
Transfusion (%) **[** a **]**	*yes*	2	7	1	
	*no*	4	37	2.64 (0.20–22.58)	0.2629
CVC (%) **[** a **]**	*yes*	4	22	1	
	*no*	2	22	2.00 (0.25–23.94)	0.6688
Median age (IQR) **[** b **]**		57 (52–72)	66 (52.5–73)	-	0.7088
Median exposure to mp-LD (IQR) **[** b **-** c **]**		29.5 (15–31)	0 (0–11)	-	***0.0102***
Overall	-	6	44	-	-

All the 50 susceptible patients enrolled in the historical cohort (i.e. patients admitted to oncohematology unit between 4 May 2006 and 21 February 2007) were included in the risk analysis. Case were all patients defined as “incident cases” while control were all patients still “susceptible” ≥6 months after their last admission to the oncohematology unit. The results of the risk analysis provided good evidence of association between the time of exposure to multi-patient lancing devicewhile admitted with the index case and the HBV infections.

**OR** = odds ration; **95%CI** = 95% confidence interval; **mp-LD** = multi-patients lancing device, **HSCT** = hematopoietic stem cells transplant; **CVC** = central venous catheter; **na** = not any, Fisher confidence levels not possible with zero count cells (all cases were exposed).

a ) 95%CI and p-value according to Fisher's exact test.

b ) p-value according to Mann–Whitney U test.

c ) This represents patients' median exposure (in days) to multi-patient lancing device while admitted with the index case and until the onset of acute hepatitis or the first positive test for HBV.

### Enhanced surveillance

Three further cases of HBsAg sero-conversion were found between March 2007 and March 2008 (2 had already been tested anti-HBcAg positive at admission and 1 had never been tested before). HBV molecular characterization was done for 2 of them as one prevalent case had a low HBV DNA load (116 UI) which did not allow typing. The results of molecular investigation showed that both patients were infected with unrelated HBV variants (CE-77 and CE-55 in figure 1).

### Audit

**Figure 3:**
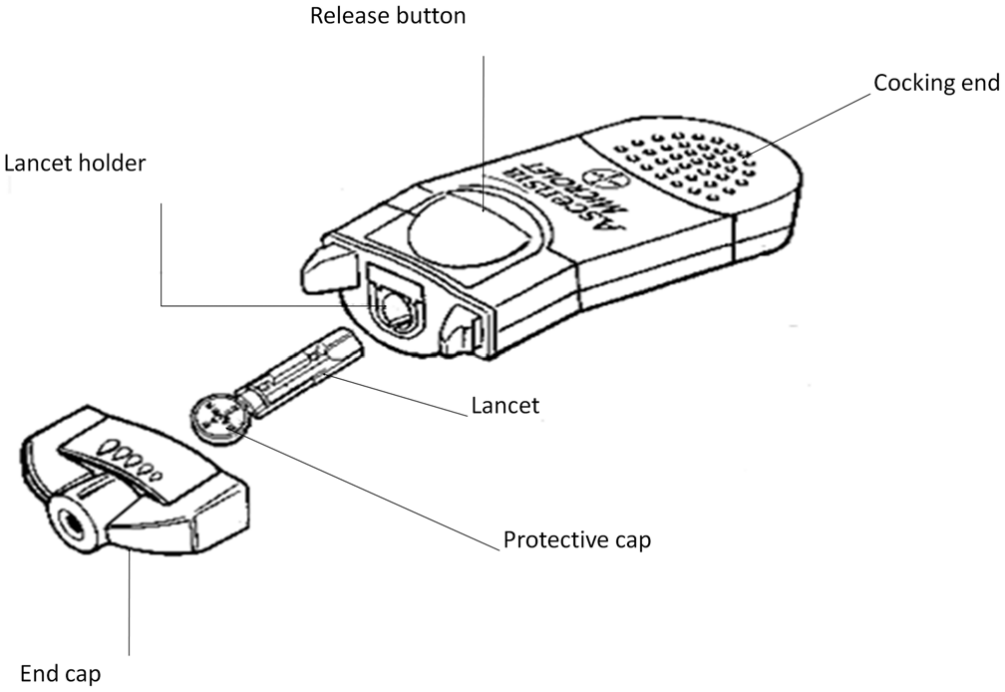
Multi-patients lancing device. Figure modified form the original as reported in the 2007 Italian version of the manual users' manual [ref. 8].

The oncohematology unit was staffed with 15 nurses on 3 different shifts; no HCW was exclusively assigned to single bed rooms. Analysis of protocol showed that no formal surveillance was implemented to screen newly admitted patients for HBV. Some nurses reported they had occasionally shared 100 ml 0.9% NaCl solutions for medication of central venous catheter insertion sites until the onset of HBV cases. Nurses claimed they consistently washed hands and used a new pair of gloves whenever they approached a new patient. All interviewed HCWs confirmed that a single shared multi-patient lancing device (figure 3) was in use, we discouraged this practice and obtained the device for testing. HCWs claimed to use the device according to the user's guide as provided by the manufacturer; we consulted the 2007 release of the user's guide [11] which reported that use of the device on multiple patients was allowed provided that both the end-cap and the lancet were changed for each subsequent patient to be sampled.

The audit in the transfusion medicine unit revealed that the unit was provided with adequate protocols for environmental cleaning and patient management. Hematopoietic stem cell aphereses/transplants were performed by transfusion medicine unit personnel at patients' beds in the oncohematology unit. The Hematopoietic stem cell were sent immediately after collection to transfusion medicine unit to be manipulated in a dedicated room provided with a fume hood, and eventually stored in another room in a liquid nitrogen cryo-tank. The apheresis machine in use was provided with single-use external circuits which prevents contact with patient's blood and the structural components. The apheresis machine was stored and maintained by nurses in a dedicated room.

The assessment of all interventional radiology unit's protocols/procedures and the informal interviews with nurse coordinator and the head of the unit confirmed that interventional radiology unit operate according to high infection control standards as required for surgical units.

The results of HCWs' tests annually performed showed that no HCW had tested HBsAg positive between 2006 and 2007 nor had they reported signs or symptoms of acute hepatitis after being tested.

### Environmental investigation

Local inspection of oncohematology unit and transfusion medicine unit confirmed all primary audit findings. According to published data and audit results we decided to search for HBV-DNA on liquid nitrogen contained in the cryo-tank and on the multi-patient lancing device which had been used in the oncohematology unit for at least 1 year. Tests of the liquid nitrogen failed to find evidence of HBV-DNA. In contrast, the analysis preformed on multi-patient lancing device showed that this device was contaminated with a HBV molecular variant identical to the one which had been found infecting both the index case and the confirmed incident cases (Figure 1).

## Discussion

The evidence collected throughout the investigation suggests that one patient, already known to be a HBsAg carrier (index case), transmitted HBV infection to 3, and potentially 6, other patients, (i.e.: 3 confirmed case and 3 suspect cases). The transmission was likely to occur as the consequence of cross infection through a shared multi-patient lancing device which was habitually used in the oncohematology to sample patients while admitted.

Several pieces of evidence support this hypothesis. Firstly, all 6 incident cases were admitted with the index case at least once between 4 May 2006 and 21 February 2007. Secondly, the index case and the 6 cases were sampled by a single multi-patient lancing device (this was the only device the nurses claim to be in use in the ward). Thirdly, the case control study showed that the median time of exposure to multi-patient lancing device while susceptible and admitted with the index case was significantly longer in cases than in controls. Fourthly, no more cases occurred after removal the multi-patient lancing device. Finally, the index case and 3 confirmed incident cases were infected with an identical HBV molecular variant which was eventually found on the multi-patient lancing device, while other coincidentally sampled cases revealed different molecular variants.

We hypothesize that the infections may have occurred by one or more of the following mechanisms: a) failure in changing the lancet, b) failure in changing the end-cap, c) infection of a new lancet occurred as consequence of contamination of the lancet holder (e.g.: blood spilling over into lancet holder; see figure 3 for multi-patient lancing device details).

Alternative transmission routes seemed to be unlikely. Transmission by transfusion was excluded since no common donor was found between cases, indeed only 2 out 6 cases had been transfused. Moreover, the risk of HBV infection through transfusion is exceedingly low in Italy due to: strict controls on the units to be transfused [12] and the progressive reduction of HBsAg prevalence in healthy adults due to compulsory anti-HBV vaccination since 1992 [13]. Potential transmission through contaminated autologous hematopoietic stem cell, as described by Tedder et al [14], was ruled out by molecular investigation on liquid nitrogen. HCW-to-patients transmission was not likely as no HCW was found to be HBsAg positive [15]. Other potential risk factors [2] such as dialysis, the use of multi-dose 0.9% NaCl vials to medicate central venous catheter insertion sites, trans-venous endomyocardial biopsy, surgery, and active drug addiction seemed unlikely on the ground of the results of risk analysis and the clonal nature of the viruses found in the incident cases and the index case. In particular, the significant association we found between HBV infection and length of exposure to multi-patient lancing device was unlikely to be affected by the potential increase of hospital staying due to acute hepatitis B. In fact, estimates were calculated according to the time a patient stayed in hospital along the index case while susceptible (i.e. until the first HBV positive test or the onset of acute hepatitis).

Although genotype D is the most frequent HBV genotype in Italy [16], genetic variability of HBV is high, due to the low fidelity of viral replication enzyme [17]. The strongest argument to support the conclusion that the index case, 3 patients and the multi-patients lancing device carried the same viral variant is that they constitute a statistically significant monophyletic cluster in the phylogenetic tree with 100% bootstrap value (obtained from 1000 replicate analysis).

The 3 cases of HBsAg sero-conversion reported during the enhanced surveillance were considered not to be part of the epidemic cluster either on the ground of pre-admission HBV status or the results of molecular typing. In our opinion they should be considered as cases of reactivation of silent HBV infection which may occur in 4%–30% of anti-HBsAg positive subjects as the consequence of severe immunosuppression for cytotoxic chemotherapy [18]–[19]. In fact, all these subjects underwent chemotherapy and 2 out 3 were known to be anti-HBcAg positive at pre-admission, while the other was proved to be infected with a different HBV molecular variant though we had no information about his pre-admission HBV status.

Our findings are consistent with other published studies which emphasize that HBV, and other blood-borne pathogens, can be transmitted by means of multi-patient lancing devices [2], [5]. The role of multi-patient lancing device in cross infection has already been suspected in a considerable number of HBV [2]–[4], HCV [20]–[21] and malaria [22] hospital outbreaks. In particular, due to its high infectivity (50% minimum infectious dose as low as 10 copies) [23] and its remarkable endurance in the environment (up to 7 days viability in dried blood) [24], HBV seem to be a top candidate for being transmitted through blood contaminated multi-patient lancing device; moreover a recent multicentre study conducted in the USA showed that multi-patient lancing devices used for capillary blood sampling in HCS may be easily contaminated with patients' blood [25].

The issue of HBV cross infection by means of multi-patient lancing device in HCS is not unique to Italy. Over the last few years similar outbreaks have occurred in other developed countries such as the USA [3], the UK [4], Germany [26], and the Netherlands [27]. This evidence highlights that patients undergoing capillary blood sampling may face an unacceptable increased risk for HBV whenever shared multi-patient lancing device are used in HCS. In fact, even when the end-cap and lancet of these devices are correctly replaced for each subsequent patient, the lancet holder can be contaminated resulting in the exposure of subsequent patients. Therefore we believe that multi-patient lancing devices should be banned from HCS and replaced with disposable safety lancets that permanently retract to prevent the use of the same device on multiple patients, as also suggested by a recent CDC guideline [28]. The use of non-disposable lancing devices should be restricted to individual use at patients' homes.

Limitations of the study are due to retrospective identification of cases, lack of a proper screening for HBV in the hematology unit and lack of biological specimens suitable for molecular typing for 3 out the 6 incident cases. All these issues may have potentially biased our results in 2 opposite directions. On one hand we might have overestimated the number of cases, as one or more of suspect cases might actually be prevalent cases who lost anti-HBcAg/anti-HBsAg through severe immunosuppression (false negative) and eventually tested positive after immune reconstitution (potential misclassification). On the other end, we might have underestimated the actual number of cases, in fact, additional case(s) belonging to the reported cluster or even whole additional cluster(s) might have passed thorough unrecognized because of the large number of patients with no information about HBV status (potential misclassification).

The results of the inferential study (case-control) might be biased because we included only patients for whom we could define the pre-admission HBV sero-status (potential selection bias). However this is quite unlikely as there is no reason for a systematic difference between exposure to risk factors and availability of pre-admission HBV sero-status. Given the small number of cases (low inferential power) we could not carry out a multivariate analysis and define other potential risk factor associated with the transmission.

Despite the above limitations this study provided, for the first time, molecular evidence to relate the use of one multi-patient lancing device to one HBV infection cluster. This evidence strongly supports the need to remove all shared pricking devices from HCS in order to avoid the occurrence of similar events.
